# Tunneling Nanotubes in Astrocyte–Neuron Crosstalk: From Intercellular Communication and Pathological Spread to Mechanobiological and Bio-Inspired Approaches

**DOI:** 10.3390/brainsci16020138

**Published:** 2026-01-28

**Authors:** Gustavo Dias, Lívia de Sá Hayashide, Bruna Pessoa, Luan Pereira Diniz, Bruno Pontes

**Affiliations:** 1 Instituto de Ciências Biomédicas, Universidade Federal do Rio de Janeiro, Rio de Janeiro 21941-902, RJ, Brazil; 2 Centro Nacional de Biologia Estrutural e Bioimagem (CENABIO), Universidade Federal do Rio de Janeiro, Rio de Janeiro 21941-902, RJ, Brazil; 3 Instituto de Educação Médica (IDOMED), Rio de Janeiro 20071-004, RJ, Brazil

**Keywords:** tunneling nanotubes, intercellular communication, neurodegenerative diseases, nanomedicine, CNS, mechanobiology, bio-inspired approaches, chemoresistance

## Abstract

Tunneling nanotubes (TNTs) are dynamic cell surface conduits that enable direct transfer of ions, signaling molecules, and organelles. They have emerged as a key mechanism of intercellular communication, complementing classical pathways such as synapses and paracrine signaling. In the central nervous system (CNS), TNTs exhibit a functional duality, particularly under aging and stress, where TNT-mediated exchange may shift from protective to maladaptive. On one hand, TNTs support homeostatic functions, ranging from mitochondrial transfer to stem cell-mediated rescue and astrocyte–neuron metabolic support. On the other hand, they facilitate the spread of prions and neurodegenerative protein aggregates, such as Tau and α-synuclein, with astrocytes playing a regulatory role. Despite rapid advances, TNT research faces challenges from conceptual heterogeneity and experimental standardization, especially in complex tissues such as the CNS. Recent mechanobiological and bio-inspired approaches, including force-based assays and three-dimensional culture models, provide new insights into TNT formation, stability, and cargo transport, extending beyond neural systems. This review offers an integrative synthesis of molecular, structural, and mechanobiological principles underlying TNT-mediated communication, emphasizing astrocyte–neuron crosstalk, while proposing validation criteria to support rigor, reproducibility, and cross-study comparability. TNTs thus emerge as dynamic, context-dependent interfaces with broad relevance to neurodegeneration, cancer, and biomedical applications.

## 1. Introduction

The human central nervous system (CNS) is composed of tens of billions of cells that exchange information through chemical and electrical signals. Among these mechanisms, synapses represent the predominant mode of communication between neurons, enabling the coordinated control of movement, sensory perception, and behavioral responses [1]. Additional pathways, including paracrine, endocrine, and autocrine signaling, involve the release of signals that stimulate receptors on the membrane of nearby cells or the cell itself [2]. Beyond synapses, astrocytes actively participate in this network, engaging in bidirectional communication with neurons through gliotransmitters, calcium signaling, metabolic coupling, and structural interactions that modulate synaptic efficacy and neural circuit function [3,4,5,6]. However, these mechanisms do not capture the full diversity of intercellular communication.

The pioneering discovery of nanometric cell-to-cell connections, named tunneling nanotubes (TNTs), was reported in 2004, when thin protrusions were first identified in rat pheochromocytoma (PC12) and Human Embryonic Kidney (HEK 293) cells [7]. These structures, with diameters ranging from 50 to 200 nm, act as bridges between cells. Unlike synapses, which mediate signaling through the release and detection of small neurotransmitters, TNTs allow the direct transfer of larger cargo, such as organelles (e.g., mitochondria and lysosomes) [7]. Given the morphological and functional overlap between TNTs and other cellular protrusions, a clear definition is essential to avoid conceptual ambiguity. Throughout this review, TNTs are structures that (i) establish direct physical contact between two non-adjacent cells, (ii) are suspended above the substratum without anchoring to the extracellular matrix, (iii) support the intercellular transfer of ions, proteins, vesicles, or organelles, and (iv) form independently of classical endocytic or exocytic pathways [7]. TNTs are primarily composed of actin filaments (F-actin), with a subset also incorporating microtubules, which confer increased stability and bidirectional transport capacity [8]. For clarity, we focus exclusively on TNTs and exclude other protrusions that lack suspended architecture or stable cell–cell contacts, including filopodia, retraction fibers, and substrate-attached extensions. This definition is applied consistently throughout the review to ensure conceptual clarity (summarized in Table 1).

Despite operational definitions, a major challenge in the field is the absence of TNT-specific molecular markers, which means that their identification often relies on morphological and functional criteria that partially overlap with other modes of intercellular communication, including extracellular vesicle (EV)-mediated transfer and transient actin-based protrusions. Such overlap complicates the experimental validation of TNT-mediated exchange, particularly in complex tissues like the CNS. Moreover, imaging-related constraints, comprising limited spatial resolution, phototoxicity, and fixation-induced artifacts, further contribute to variability across studies and can bias TNT detection and quantification. Together, these limitations highlight the need for standardized experimental strategies when studying TNT-mediated communication in both physiological and pathological contexts (summarized in Table 2).

Importantly, in the CNS, TNTs are not exclusive to neurons. Astrocytes have also been shown to form extensive TNT networks. Through these connections, astrocytes can transfer mitochondria, ions, and other cellular cargo to neurons, contributing to neuroprotection after stress or injury [9,10,11]. Beyond their beneficial roles, TNTs also participate as conduits for the spread of pathological agents [12]. In neurodegenerative disorders like Alzheimer’s disease (AD) and Parkinson’s disease (PD), TNTs provide a direct route for intercellular transfer of pathological protein aggregates, such as β-amyloid (Aβ), Tau, and α-synuclein (α-syn) [13,14,15,16]. Astrocytic TNTs appear to play a complex role in this process, potentially acting either as protective reservoirs that sequester toxic species or as active players that facilitate their propagation to neighboring cells. Importantly, this direct cell-to-cell transfer bypasses extracellular clearance mechanisms and may contribute to the progressive nature of neurodegeneration. This dual role is supported by observations that astrocytes often initiate TNT formation under metabolic or oxidative stress, forming bridges that can either rescue vulnerable neurons by transferring organelles or propagate pathological cargo to neighboring cells [17].

Despite the rapid growth of studies describing TNTs in the CNS, current knowledge remains fragmented across distinct conceptual domains. The existing literature addresses TNT biology from isolated perspectives, focusing separately on structural features, specific cargo types, individual cell populations, and/or disease contexts [18,19,20]. As a result, a unifying and comparative framework that integrates the biophysical principles governing TNT formation and stability, already observed across multiple cell types, with astrocyte–neuron crosstalk, CNS physiology and pathology, as well as emerging experimental strategies to interrogate and manipulate TNTs, is still lacking.

In this review, we address this gap by offering an integrative synthesis of TNT-mediated communication. Our goal is to bridge the mechanisms underlying TNT biology, largely characterized in diverse cellular systems, extending but not limited to neural contexts, with their functional roles in normal and pathological events in the CNS, with particular emphasis on astrocyte–neuron crosstalk. In addition, we discuss emerging experimental approaches, including mechanobiological and advanced bio-inspired models, which have been predominantly developed outside the CNS, highlighting their conceptual and methodological potential to be adapted for probing, manipulating, or even generating TNT-like structures in neural systems. Such approaches provide a forward-looking view to investigate TNTs in the CNS under physiological and pathological conditions. We also propose validation criteria to support rigor, reproducibility, and cross-study comparability. By bridging molecular, cellular, physical, and translational perspectives, this review aims to clarify conceptual boundaries and outline future research directions for TNT-mediated communication in the CNS and beyond.

## 2. Formation to Role: A Structural Perspective on TNTs and Their Implications for CNS Cells

**Figure 1:**
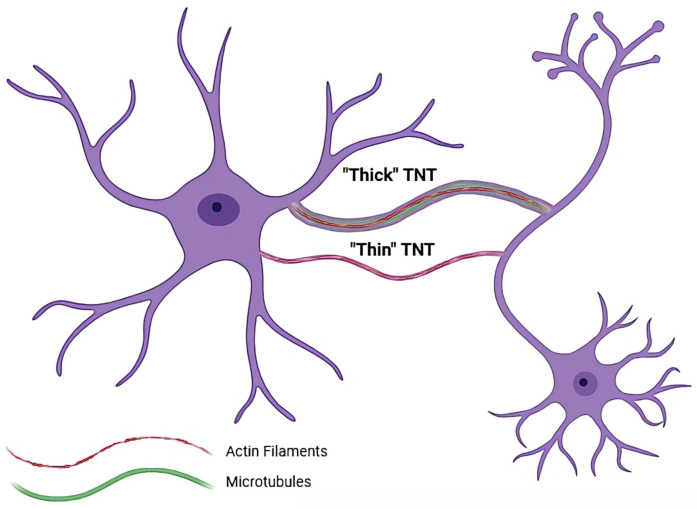
Schematic representation of the two main types of TNTs described between astrocytes and neurons. “Thick” TNTs exhibit a larger diameter and contain both F-actin and microtubules, enabling the transfer of larger cargo such as mitochondria, vesicles and other organelles. In contrast, “thin” TNTs are narrower and primarily composed of F-actin, supporting mainly the trafficking of smaller cargo and signaling molecules. These bridges mediate direct intercellular communication, contributing to metabolic and functional coordination across neural circuits.

TNT formation is a dynamic process shaped by cellular and biophysical mechanisms, which remain under active investigation. Their biogenesis varies according to cell type, physiological state, and environmental cues [21]. Two main mechanisms of TNT formation have been described (Figure 1). The first, designated Type I, is characterized by an active protrusion driven by the polymerization of actin, resembling growing filopodia that extend from donor cells to reach recipient cells [22]. A key mediator in this process is the calcium-binding protein S100A4, which acts as an extracellular attraction cue. Under stress, p53 activation triggers caspase-3-mediated cleavage of intracellular S100A4, generating a concentration gradient that guides TNT protrusion toward the target cell [17]. The second mechanism, Type II, occurs when previously contacting cells move apart, stretching the membrane into a tubular connection [21]. Although these two mechanisms can occur independently, they often coexist [21]. Type II TNTs tend to be longer, more stable, and exhibit distinct vesicle dynamics, with vesicles remaining stationary within the nanotube [23].

The cytoskeletal composition of TNTs is important for their formation and stability. F-actin is a nearly universal component of TNT biogenesis [24], and actin inhibitors such as latrunculin and cytochalasin may substantially reduce TNTs [7]. Furthermore, the cytoskeletal composition can be more complex than previously thought, with the observation of TNTs containing not only F-actin, but also intermediate filaments, formed by cytokeratin 7, and microtubules, formed by tubulin [8,25].

TNTs are not a structurally homogeneous class of intercellular conduits; rather, they can also be classified into two distinct subtypes based on their cytoskeletal composition, which dictates their stability and functional capabilities. These subtypes are generally referred to as “thin” and “thick” TNTs [26]. Notably, these TNT subtypes have distinct cellular origins and functional roles in the CNS, particularly concerning astrocyte–neuron interactions.

Thin TNTs are composed exclusively of F-actin. This composition is associated with structures that are typically more delicate and transient. Functionally, the transport of cargo through thin TNTs appears to be less efficient and predominantly unidirectional. These F-actin-only structures are more frequently observed forming from healthy cells, such as astrocytes. In contrast, thick TNTs are characterized by a more complex cytoskeleton containing both F-actin and microtubules. The presence of microtubules confers greater stability to these structures, making them less transient than their thinner counterparts [23]. Functionally, thick TNTs appear to mediate more effective transport and, critically, are capable of mediating bidirectional transfer of cargo between connected cells. In pathological contexts, such as stressed neurons or glioblastoma (GBM), these thick TNTs facilitate the intercellular spread of organelles and pathological protein aggregates, which may impact astrocytes and neurons alike [27,28].

TNT formation is a dynamic process influenced by the interplay of active cytoskeletal forces and passive membrane biophysics. The stability of these tubular protrusions depends not only on membrane bending energy but also on the lateral segregation of anisotropic membrane components. Proteins of the inverse Bin–Amphiphysin–Rvs (I-BAR) domain family and specialized membrane nanodomains possess intrinsic spontaneous curvature, which drives their preferential accumulation on highly curved surfaces, such as TNTs [29]. This curvature-induced accumulation lowers the system’s free energy and mechanically stabilizes the nanotube. Direct mechanistic support for this principle comes from optical tweezers (OT)-based studies in which membrane tethers (artificial nanotubes with dimensions similar to TNTs) are extracted from living cell surfaces, providing a controlled platform to probe the mechanics and remodeling of highly curved membrane structures [30,31,32], even in CNS cells [33,34]. Using this approach, it has been shown that membrane tethers do contain F-actin, as in TNTs [31,33]. These findings demonstrate that nanotube stabilization emerges from a dynamic coupling between membrane curvature and cytoskeletal remodeling [30,31].

In astrocytes, this stabilization facilitates metabolic support and organelle transfer to neurons, while in neurons it promotes the delivery of mitochondria and signaling molecules back to astrocytes, reinforcing bidirectional communication [35]. Simultaneously, active forces from actin polymerization exert outward pressure on the membrane, possibly driving TNT protrusion. The combination of these active and passive mechanisms enables the formation and elongation of TNTs as stable structures [36,37].

A positive feedback model proposes that a small fluctuation in membrane shape attracts curvature-activating proteins, which in turn recruit and intensify actin polymerization, pushing the membrane outward in a cycle that amplifies the protrusion [38,39]. Proteins such as Myosin-X act as key regulators in this process, generating the required force to pull and elongate TNTs along cytoskeletal tracks [40]. Finally, TNT formation can be influenced by a series of external stimuli. In the CNS, astrocytes often initiate TNTs under metabolic or oxidative stress, forming protective bridges toward unstressed neurons [41]. The formation appears to increase under conditions of cellular stress, such as metabolic stress (nutrient deprivation), environmental stress (exposure to hydrogen peroxide or hypoxia), and inflammation. The observation that TNTs form between stressed and non-stressed cells suggests their dual role, potentially propagating pathology while also mediating cellular rescue, transporting organelles and other materials to cells in danger [42,43,44,45].

## 3. Mechanobiological Approaches to Study TNTs

As previously mentioned, the dynamic interplay between active cytoskeletal forces and passive membrane biophysics appears to govern TNT formation, highlighting their role as mechanically and functionally specialized conduits. Accordingly, their stability cannot be fully understood without quantitative analyses of the forces that shape their behavior. Mechanobiological studies have begun to provide this insight, linking TNT structure to function, testing their resilience under mechanical stress, and measuring cargo-transfer efficiency. By integrating molecular with mechanical perspectives, these emerging strategies offer a framework to interrogate TNT formation, stability, and function, illustrating how physical manipulation can complement molecular and structural insights. This section reviews works using OT, atomic force microscopy (AFM), micropipette-based manipulation, and other related approaches, illustrating how mechanobiology can directly or indirectly probe TNTs. Although many quantitative mechanobiological approaches to TNTs were initially developed in non-neural cellular systems, the physical principles they reveal are directly relevant to the CNS.

Pioneering studies on the mechanical properties of TNTs began with Pontes et al. [46], who used OT to characterize TNTs formed between GBM cells (U-87MG cell line) in two-dimensional (2D) cultures. The study provided one of the first quantitative measurements of TNT elasticity, indicating that these structures are force-bearing, with TNTs capable of undergoing deformation without rupture and exhibiting characteristic bifurcations under applied force [46]. Building on these findings, Patheja and collaborators [47] applied OT in three-dimensional (3D) tumor spheroids derived from a human oral squamous cell carcinoma (patient-derived cell line). Despite the different tumor type, TNTs were actively deformable while maintaining vesicle and molecular transfer, highlighting mechanical robustness in more complex 3D contexts, particularly at the spheroid periphery [47]. Subsequent studies expanded mechanobiological investigation to other cell types and tools. Li et al. [48] applied AFM to assess TNTs formed by HEK 293 cells, quantifying their stiffness and confirming that they can bear mechanical load, while also showing how external forces influence their stability and cargo-transfer capability. Sun and collaborators [49] employed micropipette-based manipulation to examine TNTs formed by myoblasts (C2C12 cell line) and HEK 293 cells, generated by different mechanisms of formation. They demonstrated that mechanical properties were independent of the mode of formation (Type I or Type II), but their stability and force-bearing capacities are closely linked to their cytoskeletal composition. Wang et al. [50] also used micropipette-based manipulation to directly deform TNTs connecting rat kidney epithelial cells (NRK cell line), demonstrating that the conduits can transmit mechanical signals between connected cells, induce calcium influx, and alter the visco-mechanical state of the recipient cell, revealing a direct link between TNT-mediated mechanical force and cellular response.

Beyond probing pre-existing TNTs, mechanobiological approaches have also been applied to generate TNT-like structures [51,52,53] using a range of techniques including micropipette-based manipulation, OT, electrical stimulation, or synthetic lipids. These approaches provide a unique opportunity to decouple biochemical signaling from physical forces, allowing direct interrogation of the mechanical principles underlying TNT formation and function. This idea dates back to the pioneering work by Orwar and colleagues, who demonstrated that giant artificial vesicles can form networks of nanotubes, allowing membrane fusion and molecular transfer between compartments in fully artificial systems [54,55,56,57]. Related studies further showed that similar tubular connections can mediate DNA and RNA exchange between protocells, suggesting that nanotube-mediated communication may represent a primitive mechanism of intercellular interaction [58]. Building on these concepts, studies in living cells demonstrated that stable TNT-like protrusions can be artificially generated and functionally engaged in intercellular communication. For example, Pascoal et al. [51] used OT to pull membrane nanotubes between cells, suggesting that these conduits appear capable of transmitting electrical signals over long distances. Zhang and collaborators [52] created artificial TNT-like connections using micropipette-based manipulation between cells, demonstrating directional intercellular transfer of small molecules and ions, including calcium and fluorescent enzymatic substrates. Kozintsev and Sugihara [53] developed synthetic lipid nanotubes to form TNT-like bridges between HeLa cells, allowing passive molecular transport and illustrating how lipid composition can influence tube properties and membrane fusion.

Altogether, these studies demonstrate that TNTs are mechanically active, force-bearing structures whose formation, stability, and function are tightly coupled to physical cues. Mechanobiological approaches have revealed that TNTs can be generated, remodeled, or deformed without rupture and are capable of transmitting signals across 2D cultures, 3D tumor spheroids, and artificial systems. Although most investigations to date have focused on non-neural systems, they establish an experimental and conceptual framework that can be extended to interrogate TNT mechanics and function in neural contexts. Importantly, the principles uncovered—mechanical robustness, force-induced generation, and cytoskeleton-dependent stability—can offer experimental directions applicable to astrocyte–neuron systems, where calcium signaling, metabolic coupling and stress-induced remodeling are central features. Mechanobiological strategies thus emerge as powerful experimental perspectives to probe TNT behavior under physiologically and pathologically relevant conditions in the CNS.

## 4. TNTs as Bioenergetic Support Platforms in Cellular Stress and Regeneration

Beyond revealing TNTs as force-bearing conduits, mechanobiological studies also offer a conceptual/mechanistic perspective to explore how these structures sustain intercellular rescue under stress. The same physical properties that allow TNTs to withstand deformation, transmit forces, and maintain stability over long distances are fundamental to their role as platforms for cargo exchange. Accordingly, one important biomedical implication of TNTs appears to lie in their ability to act as a rescue system in response to cellular stress, including hypoxia, changes in glucose concentrations, and pH levels [43], particularly in metabolically demanding tissues such as the CNS, where cells are extremely sensitive to energy imbalance.

The main mechanism that underlies the beneficial effects of TNT formation is the mitochondrial transfer between cells. The intercellular transfer of mitochondria is a complex phenomenon in which TNTs are increasingly considered as one of the main platforms for these exchanges, which can be unidirectional or bidirectional. The transport of organelles through these structures is often facilitated by microtubules, with thick TNTs being associated with long-distance mitochondria transport, while thin TNTs are used for short distances [59,60]. The direction of transfer is often context-dependent, typically from healthy to damaged cells, although in certain cases mitochondria may move from malignant to non-malignant cells [61]. This exchange appears to have significant functional consequences for the recipient cells, such as the rescue from apoptosis, restoration of mitochondrial function, increased proliferation and invasion of cancer cells, and acquisition of chemoresistance [62].

**Figure 2:**
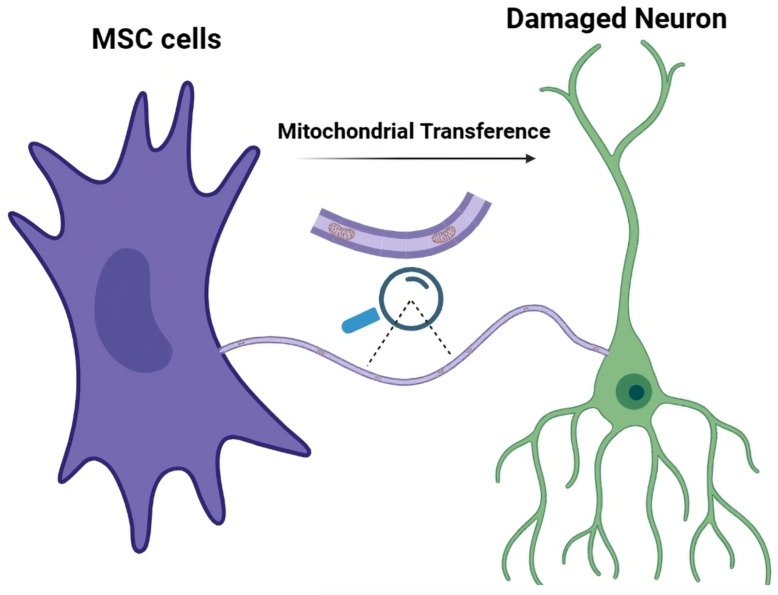
Schematic representation of TNT-mediated mitochondrial transfer from MSCs to a damaged neuron. Healthy Mesenchymal stem cells (MSCs) form TNTs that allow the directed movement of functional mitochondria into stressed neuronal cells. This intercellular transfer supports bioenergetic recovery, reduces oxidative stress and promotes neuronal survival under injury conditions.

A striking example of TNT-mediated protection is found in mesenchymal stem cell (MSC)-based regenerative therapies, represented in Figure 2 [63]. This response aligns with the stress-induced TNT formation mechanisms described earlier, highlighting how physical and metabolic cues converge to promote intercellular rescue. When exposed to stress signals from cells injured by ischemia, oxidative damage, or inflammation, MSCs form membrane bridges to promote cellular rescue [64]. The most documented protective effect is the transfer of healthy mitochondria, which appears to decrease the levels of reactive oxygen species (ROS), and inhibit apoptosis [65]. In neural systems, this mechanism extends to interactions between MSCs, neurons, and glial cells, where TNT-mediated mitochondrial transfer preserves neuronal survival and glial support functions under stress [66]. This rescue mechanism shows promising efficacy in a wide range of preclinical models (ischemia, reperfusion), supporting TNTs as important mediators in regenerative actions [66].

Beyond energetic restoration, MSCs also transfer other cargos through TNTs [67,68]. In mouse models of myocardial infarction, MSCs were shown to transport soluble growth factors such as the hepatocyte growth factor (HGF) and the vascular endothelial growth factor (VEGF), potentially promoting angiogenesis and influencing the immune response through cytokine exchange that reduces inflammation [67]. Moreover, the formation of these connections is also facilitated by the gap junction protein Connexin-43 [68]. This complex and regulated communication pathway underscores the emerging roles of TNTs in cell therapies and points to their potential as a target for future strategies in regenerative medicine. Intriguingly, emerging studies indicate that TNT dynamics undergo substantial remodeling during aging, although whether these changes represent an adaptive response or a detrimental shift remains unclear. These age-associated changes may critically influence both the efficiency of intercellular rescue and the susceptibility to pathological spread, particularly in the aging CNS.

## 5. Astrocytic TNTs in Aging and Senescence: From Neuroprotection to Neurodegeneration

While the last section highlights TNTs as robust platforms for bioenergetic rescue and mitochondrial transfer under acute cellular stress, aging introduces a distinct and more complex context in which these same mechanisms may become impaired, maladaptive, or even detrimental. Aging is a progressive, multifaceted process marked by the gradual decline of cellular resilience and tissue homeostasis, ultimately increasing vulnerability to dysfunction and disease [69]. In the CNS, the accumulation of senescent astrocytes is a major driver of brain aging, as these cells exhibit mitochondrial dysfunction, elevated oxidative stress, and impaired proteostasis, compromising their essential roles in metabolic support, neurotransmitter regulation, and neuroinflammation control, thereby increasing neuronal vulnerability and accelerating degenerative processes [70]. In this context, TNTs represent an important mechanism of intercellular communication whose remodeling during aging may further exacerbate astrocyte dysfunction and contribute to the decline of neural circuit integrity.

Astrocytes are essential for synaptic metabolic support, clearance of extracellular debris, antioxidant protection, and regulation of neuronal excitability [71]. TNTs extend these roles by enabling the directed transfer of bioenergetic substrates and healthy mitochondria to injured neurons, potentially restoring adenosine triphosphate (ATP) production and reducing apoptosis [19]. Although direct evidence is still lacking, several indirect findings are consistent with the hypothesis that TNT formation could increase with aging and cellular senescence, at least transiently, as part of a compensatory response: senescent astrocytes exhibit enhanced cytoskeletal remodeling [72], elevated mitochondrial stress [73], and increased ROS production [73], as well as persistent inflammatory signaling through the senescence-associated secretory phenotype (SASP) [74]. These alterations are known to promote cell surface protrusion dynamics and can facilitate the initiation of thin TNTs. Furthermore, the aged brain is characterized by impaired mitochondrial homeostasis [73] and diminished astrocyte-mediated metabolic support, which may activate compensatory mechanisms aimed at boosting organelle transfer.

At the molecular level, astrocyte senescence engages signaling pathways that are already known to regulate TNT biogenesis in response to stress. Under physiological contexts or early stress conditions, TNT biogenesis in astrocytes is stimulated by p53 activation, oxidative cues, and metabolic demands, acting as a neuroprotective rescue mechanism [41]. It was recently demonstrated that human senescent astrocytes exhibit increased p53 levels [74], consistent with previous reports showing activation of SASP in glial cells [75], suggesting that elevated p53 may contribute to cytoskeletal remodeling and favor the formation of TNTs, as observed in other stress conditions where senescent or reactive astroglia rely on intercellular connections to mitigate damage [14]. α-syn fibrils induce a senescent phenotype in astrocyte cultures [76], reinforcing that synucleinopathy-related stress accelerates cellular aging. α-syn-induced senescence promotes the transient nuclear localization of focal adhesion kinase (FAK), a key cytoskeletal regulator [10]. FAK-dependent regulation of Rho-associated kinases plays a central role in TNT formation and stabilization, suggesting that dysregulation of this pathway may underlie altered TNT biogenesis during astrocyte senescence. In parallel, senescent astrocytes respond to ROS accumulation by increasing TNT formation to expel damaged organelles and reestablish redox homeostasis, a process that can ultimately reverse senescence [5]. Given the α-syn intrinsic toxicity, including mitochondrial dysfunction and energetic collapse [77], characterizing TNT dynamics in well-established senescence models is important to dissect the specific contribution of the senescence program to changes in intercellular communication.

Previous findings demonstrate that senescent murine astrocytes accumulate damaged mitochondria [73], characterized by increased activation of the fission machinery [78], impaired mitophagy, and excessive production of mitochondrial ROS [73]. These alterations compromise mitochondrial quality and likely limit their ability to engage in TNT-mediated mitochondrial transfer, since dysfunctional organelles represent a detrimental cargo for recipient cells. It was also shown that selective removal of damaged mitochondria restores homeostasis and reverses SASP, reinforcing that mitochondrial quality control likely influences astrocyte intercellular support through TNTs. When mitochondrial donation becomes insufficient, the cargo polarity may invert: instead of delivering high-quality mitochondria, senescent astrocytes offload damaged organelles and dysfunctional vesicles, thereby amplifying neuronal metabolic distress [79].

Senescent cells develop a SASP characterized by persistently elevated secretion of inflammatory cytokines, chemokines, growth factors, and proteases, typically including the interleukins IL-6, IL-8, IL-1β, and the tumor necrosis factor (TNF-α) [74,80]. This sustained SASP activity raises TNF-α, IL-1β, and IL-6 levels, which may increase ROS production, destabilizing TNT architecture and impairing membrane curvature, although TNF-α is known to promote the formation of TNTs in certain cells [81].

Mitochondria become the primary source of ROS in senescent cells [82], establishing a feed-forward loop where excessive ROS further impairs mitochondrial quality control, disrupts mitophagy, and worsens bioenergetic decline [61]. While acute ROS bursts may transiently promote TNT formation, chronic oxidative stress decreases their stability and transport efficiency, leading to shorter nanotube lifespans and erratic, poorly regulated cargo transfer between cells [5,83].

Biomaterial engineering has begun to leverage this mitochondrial stress response to intentionally stimulate intercellular mitochondrial exchange via TNTs; for example, studies using engineered nanoparticles demonstrated that controlled mitochondrial damage increases ROS and activates the PI3K/AKT/mTOR pathway, which may enhance TNT formation and support the transfer of healthy mitochondria from donor to injured cells, a strategy being investigated as a therapeutic approach [84]. Modulating TNT-mediated intercellular communication may offer an anti-aging therapeutic strategy: in an MSC spheroid model, functional TNTs enabled the transfer of cytoplasmic components from low-passage to high-passage cells, reducing senescence markers and improving mitochondrial and lysosomal function [85]. Sirtuin 1 (SIRT1) is a well-established longevity-regulating enzyme [86], and a recent study shows that its activation may counteract arsenic-induced mitochondrial dysfunction and cellular senescence by preserving F-actin organization, sustaining TNT formation, and maintaining intercellular mitochondrial transfer, ultimately protecting liver cells [87].

These findings suggest a mechanism through which SIRT1 combats cellular aging, highlighting the potential relevance of TNT-mediated mitochondrial exchange. Finally, engineered nanotube topographies illustrate that cellular aging reversibility is not driven only by the exchange of cytoplasmic components through intercellular nanotubes but also by mechanical cues: synthetic TiO_2_ nanotubes restore function in senescent MSCs by activating mechanotransduction pathways such as the Yes-associated protein (YAP) and improving structural integrity, mitochondrial performance, and differentiation capacity [88].

These advances show how mechanobiology and nano-engineering can influence senescence through both biochemical communication and biomechanical reinforcement. Future studies will need to determine how these mechanisms can be optimized to design new therapeutic strategies to combat senescence in the CNS, including approaches aimed at rejuvenating aged astrocytes and preserving brain homeostasis. Notably, TNT remodeling may act as a significant contributor to neurodegenerative disease progression.

## 6. Dual Roles of TNTs in Neurodegenerative Disease Progression

Building on the age- and senescence-associated remodeling of TNTs discussed above, this section examines how the same intercellular routes that enable metabolic rescue can be repurposed to propagate pathology in neurodegenerative disease. In this context, TNTs emerge as context-dependent conduits whose impact is dictated by cellular state, cargo identity, and network organization within the CNS.

**Figure 3:**
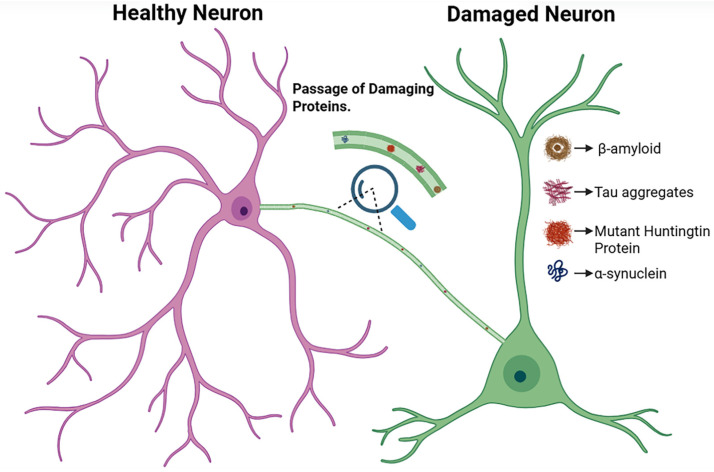
Schematic representation of the pathogenic roles of TNTs. While TNTs may contribute to neuroprotective processes under certain conditions, they can also facilitate the intercellular transfer of disease-associated agents. Stressed or damaged neurons may use TNTs to transmit pathological cargos, including β-amyloid, Tau aggregates, mutant huntingtin protein, and α-synuclein aggregates, to neighboring cells. This process may contribute to the spread of neurodegenerative pathology and infectious agents within neural networks.

AD emerges primarily due to the accumulation of Aβ plaques and hyperphosphorylated Tau protein tangles in the brain, leading to synaptic dysfunction, neuronal loss, and progressive cognitive decline [89]. Mitochondrial impairment is a central feature of AD pathophysiology, characterized by increased ROS, oxidative damage to cellular components, energy failure, and apoptotic cell death [90]. In parallel, activation of microglia and astrocytes amplifies neuroinflammation through the release of cytokines, further accelerating disease progression [91]. Within this pathological landscape, TNTs have been reported to play a neuroprotective role by mediating the transfer of healthy mitochondria from donor cells, such as neurons, neural stem cells, mesenchymal stem cells, and astrocytes, to metabolically compromised neurons [19,92]. This TNT-mediated mitochondrial transfer restores ATP production, reduces oxidative stress, and lowers apoptotic rates, resulting in functional improvement in experimental AD models [93,94]. However, the same TNT networks that support metabolic rescue can also facilitate the intercellular dissemination of pathological aggregates. Both Tau and Aβ have been shown to propagate directly between cells through TNTs, particularly under stress conditions such as oxidative imbalance or nutrient deprivation [41,92,95]. This duality illustrates a central paradox in AD: TNTs can simultaneously support neuronal survival and contribute to the spatial spread of toxic protein species (Figure 3).

Prion diseases represent another clear example of TNT-mediated pathology. These disorders are caused by the conformational conversion of the normal cellular prion protein into its pathogenic scrapie isoform [96]. TNTs provide an efficient route for the direct dissemination of prions between neurons and neuroimmune cells, supporting spread within the CNS and potentially from peripheral tissues into the brain [97,98]. Two exclusive modes of TNT-mediated prion transfer have been described: surface-bound transport of GPI-anchored prion proteins along the external membrane of TNTs [99], and vesicular transport through the nanotube lumen, involving endosomal, lysosomal, and recycling compartments [100,101]. These observations position prion diseases as a paradigmatic case illustrating how TNTs can bypass extracellular clearance mechanisms and promote long-range propagation of misfolded proteins.

In Huntington’s disease (HD), expansion of CAG repeats in the huntingtin gene results in the production of mutant huntingtin (mHTT) proteins that form toxic intracellular aggregates. The anatomical progression of HD suggests a prion-like dissemination mechanism. TNTs also have emerged as an important pathway in this process, enabling direct, contact-dependent transfer of mHTT aggregates between neuronal cells, particularly within striatal circuits [102,103,104]. A distinctive feature of TNT-mediated transport in HD is its molecular selectivity. The striatum-enriched GTPase Rhes actively induces the formation of TNT-like protrusions that preferentially transport mHTT and other polyglutamine-expanded proteins, but not wild-type huntingtin or unrelated aggregation-prone proteins such as Tau [105]. mHTT is transported within endosomal and lysosomal vesicles, underscoring the regulated and active nature of this intercellular exchange.

Similarly, PD progression is tightly linked to the spread of α-syn aggregates that form Lewy bodies and neurites [106]. α-syn has been shown to traffic through TNTs predominantly within lysosomal vesicles [15]. Importantly, α-syn transfer establishes a positive feedback loop: internalization of aggregates increases intracellular ROS levels, which in turn promotes further TNT formation, accelerating pathological spread. Astrocytes contribute to this process by redistributing α-syn via TNTs within the glial network, relieving local stress at the cost of amplifying pathology at the tissue level [14].

Finally, and in contrast to neurodegenerative disorders, where TNTs link degeneration and repair, their role in brain tumors is predominantly associated with adaptation and therapeutic resistance. In gliomas and GBM, TNT formation is strongly enhanced by cellular stress and exposure to chemotherapeutic agents. These tumors develop extensive TNT-based communication networks that connect cancer cells (homotypic interactions) as well as cancer and stromal cells (heterotypic interactions). Through these networks, tumor cells exchange metabolites, signaling molecules, and organelles, collectively enhancing survival and resistance to standard-of-care therapies [107]. This TNT-mediated integration of the tumor microenvironment represents a distinct functional regime in which intercellular connectivity directly supports malignancy, underscoring the context-dependent nature of TNT biology in the CNS.

## 7. The Nanomedicine Potential of TNTs: A Double-Edged Interaction

Building directly on the dual roles of TNTs described in the previous section—where these structures support neuronal survival while simultaneously enabling the spread of pathology—nanomedicine emerges as a particularly relevant and timely framework to explore this ambivalence. Moreover, in the CNS, where therapeutic efficacy is constrained by the blood–brain barrier and the complex organization of astrocyte–neuron crosstalk, TNT-mediated intercellular transport introduces an additional and largely underexplored variable that can profoundly influence drug biodistribution, efficacy, and neurotoxicity.

Nanomedicine represents an emerging approach in therapeutics, utilizing nanoscale delivery systems, collectively termed nanomedicines (NMeds), to overcome the limitations of conventional therapies [108]. These NMeds are engineered to optimize the treatment of a wide range of pathologies, including neurodegenerative diseases, that require crossing the blood–brain barrier [109], and cancer, through advantages such as enhanced solubility, protection of sensitive payloads (like genetic material and proteins) from degradation, controlled release, and, most importantly, precise targeting of specific cells or tissues [110,111,112,113,114]. However, as highlighted for pathogenic proteins and organelles in neurodegeneration, the efficacy and final fate of these advanced systems are influenced by an increasingly relevant participation of TNTs. In the CNS, TNT-mediated transport appears to add an additional layer of complexity to nanomedicine, influencing both therapeutic efficacy and off-target neurotoxicity.

The crosstalk between NMeds and TNTs opens new avenues for drug delivery studies. On one hand, the ability of TNTs to transport NMeds between cells represents a major challenge for targeted therapy; an NMed that reaches its target cell can be subsequently redistributed to healthy neighboring cells [108], minimizing its therapeutic effect and potentially increasing off-target toxicity. On the other hand, this same transport pathway offers potential opportunities. TNTs can be exploited as biological highways to improve the biodistribution of NMeds throughout diseased tissues, such as solid tumors or the pathological brain, multiplying the reach and efficacy of the treatment [108].

3D-bioprinted cancer models, which allow for the formation of TNTs in a controlled microenvironment, are becoming powerful tools to investigate these complex interactions and their impact on drug susceptibility [115]. Consequently, the modulation of TNTs—whether by inhibiting their formation to contain the spread of pathologies or drugs, or by stimulating them to improve therapeutic delivery—emerges as a new and promising target for the future of nanomedicine. Understanding how NMeds interact with TNT networks is relevant for several tissues, but particularly important in the CNS, where unintended redistribution between neurons and glial cells may shift therapeutic outcomes from neuroprotection to neurotoxicity.

Early studies using inorganic nanoparticles provided the first direct evidence that TNTs can function as intercellular transport routes for NMeds, both in vitro and in vivo. This evidence emerged from pioneering studies, which directly visualized this intercellular exchange. Quantum Dots (QDs) [116] were observed moving between cells within vesicular compartments such as lysosomes, revealing a key structural distinction [22,117]: bidirectional cargo transport was an exclusive capability of thick TNTs, in contrast to thin TNTs [117]. Follow-up work using fluorescent nanodiamonds expanded the scope of this phenomenon, proving that the transfer is not limited to cells of the same type (homotypical) but also occurs efficiently between cells of different types (heterotypical) [118]. The physiological relevance of these findings was solidified with the observation of QD transport between macrophages in vivo in the muscle tissue of mice, confirming that communication via TNTs is not an artifact of cell cultures [119]. Furthermore, studies with mesoporous silica nanoparticles demonstrated that the transfer rate can be externally modulated, being increased by cellular stress and decreased by hyperthermia, which underscores the potential to control this delivery pathway for therapeutic purposes [120].

Perhaps the most significant findings stem from studies using targeted, biodegradable poly(lactic-co-glycolic acid) (PLGA) decorated with the g7 peptide nanoparticles designed for CNS delivery. These studies not only confirmed homotypical transfer but also documented crucial heterotypical transfer from glial cells to neuronal cells, suggesting a novel route to access traditionally hard-to-target neurons. Critically, this process was actively enhanced; inducing an increase in TNTs via the mSec protein resulted in a nearly 25% increase in the transport of NMeds to neurons. These findings also imply that glial cells can function as biological intermediaries for nanoparticle distribution, transferring therapeutic cargo toward neurons through TNTs. Such glia-to-neuron communication opens a promising route for treating neurodegenerative diseases that involve mitochondrial dysfunction or impaired intracellular trafficking. Moreover, the findings also establish that the interplay between polymeric NMeds and TNTs is a dynamic and controllable process, highlighting the therapeutic potential of modulating TNTs to either localize or enhance drug delivery [121,122].

The interaction between lipid nanoparticles and TNTs constitutes a complex field of investigation, revealing that these NMeds are not only transported by these pathways but can also actively modulate their biogenesis and structural typology [108]. Pioneering studies with solid lipid nanoparticles demonstrated that their administration to cells, although non-neural, induces an increase in the formation of TNTs, which underscores the stress effect that NMeds can exert and the consequent adaptation of the intercellular communication network [123]. This modulation can be exploited for therapeutic purposes.

Such sophisticated interactions are more evident in GBM models. The administration of doxorubicin-loaded liposomes to GBM cells, which tend to form thick TNTs, induces a phenotypic switch, leading to the predominant formation of thin TNTs, whose cargo transport properties are lower and more similar to those formed by healthy astrocytes. Even more significantly, the use of targeted liposomes (decorated with the apolipoprotein E and chlorotoxin) in mixed cultures revealed a preferential directionality of transport. The transfer via TNTs appears more frequent between cells of the same type (homotypical GBM-GBM transfer) than between different cell types (heterotypical GBM-astrocyte transfer). This discovery is of great importance, as it suggests a new therapeutic paradigm in which the targeted delivery of liposomal drugs could be optimized by homotypical transport, increasing the biodistribution of the drug within the tumor population while minimizing its toxicity to surrounding healthy cells [124,125,126,127].

In relation to the application of nanotechnology engineering in TNTs, a study was developed in 2021, emphasizing the recent advances in scientific models in TNTs research [115]. This was achieved through the development of 3D cancer models using bioprinting technology. With the aim of overcoming the limitations of 2D cell cultures and the hypothesis that TNTs could be an artifact of these artificial conditions, a proof-of-concept study was developed. The methodology involved the formulation of an optimized bioink, composed of a hydrogel of collagen, sodium alginate, and gelatin, designed to mimic TME. Renal cancer cells from the 786-0 cell line were directly incorporated into this bioink, which was then used to print, layer by layer, a 3D scaffold, allowing for the investigation of intercellular communication in a controlled and reproducible environment [115]. The results demonstrated that the bioprinted cells not only maintained high viability (approximately 90%) and proliferative capacity for long periods (at least 15 days), but were also able to actively build long and thin cellular projections, morphologically and functionally similar to TNTs, within the 3D matrix. The functionality of these structures was supported by the observation, through time-lapse microscopy, of the intercellular transport of mitochondria along these channels, with a migration rate (approximately 33 µm/h) similar to those observed in 2D models [115]. Thus, the formation of TNTs is a process that cancer cells carry out autonomously within a complex 3D microenvironment, which provides strong evidence that these structures are not an artifact of 2D cultures. The study, therefore, establishes that 3D bioprinting technology is a powerful and validated platform for the future investigation of the role of TNTs in cancer progression, the acquisition of drug resistance, and other pathological processes, offering a more physiologically relevant model for scientific research [115]. Extending these NMeds–TNT interactions to CNS-relevant 3D models will be essential to predict therapeutic performance in the brain, where cellular heterogeneity and long-range communication are defining features.

## 8. Limitations and Outstanding Challenges

Despite significant progress, the study of TNTs still faces important technical and conceptual limitations. A major challenge is the absence of TNT-specific molecular markers, which complicates their identification and hampers selective genetic or pharmacological manipulation. Recent efforts have begun to address this gap, including a comprehensive proteomic survey of TNTs, referred to as the TNTome [128]. However, candidates remain largely correlative and have not yet yielded validated markers suitable for routine detection. In addition, TNT-mediated transfer partially overlaps with EV-based communication pathways, making it difficult to fully disentangle contact-dependent exchange from vesicle uptake, particularly in complex environments. Imaging-related constraints also remain critical, as TNTs are highly dynamic, submicron structures that are sensitive to phototoxicity, fixation artifacts, and segmentation bias, especially in thick or heterogeneous samples. Finally, most mechanistic insights into TNT biology derive from in vitro systems, where substrate properties, cell density, and stress conditions may influence TNT formation. Translating these observations to in vivo contexts, such as the CNS—characterized by dense tissue architecture, cellular diversity, and limited optical accessibility—remains a major challenge. Addressing these limitations requires the development of standardized validation criteria, improved imaging strategies, and physiologically relevant 3D and in vivo models. In this context, and considering the current state of the field, we propose a set of TNT validation criteria (Table 1) and a comparative framework of experimental strategies (Table 2) to support rigor, reproducibility, and cross-study comparability in TNT research, especially within the CNS.

## 9. Conclusions

The biology of TNTs reveals a striking functional duality. On one hand, these structures are essential for cellular repair and survival, exemplified by the transfer of healthy mitochondria to metabolically compromised neurons, which restores bioenergetic function and limits cell death in neurodegenerative contexts [93,94]. Similarly, MSC-based therapies rely on TNTs to mediate bioenergetic rescue and delivery of growth factors, reinforcing their role in regenerative processes [63,68]. On the other hand, the same properties that make TNTs vital for cellular repair also render them vulnerable to pathological exploitation. TNTs function as conduits for the propagation of toxic protein aggregates, such as Aβ and Tau in AD, α-syn in PD, and mHTT in HD, allowing the pathology to spread predictably and efficiently throughout the entire neural network [13,14,15,16,95,102].

The broader relevance of TNTs lies in their ability to mediate the transfer of complex cargos, such as complete organelles, protein aggregates, viral particles, or nanomedicines, in a way that other communication pathways, like synapses, cannot [7]. Notably, mechanobiological approaches have revealed that TNTs are not passive conduits but mechanically active structures, with formation, stability, and function tightly regulated by physical forces, cytoskeletal dynamics, and cellular tension. Such a perspective places TNTs at a conceptual crossroads between mechanobiology, which seeks to define the physical principles underlying their formation and resilience, and nanomedicine, which seeks to harness these structures for therapeutic benefit.

The integration of advanced experimental platforms, including in vitro 3D and bioprinted tissue models, offers powerful opportunities to dissect TNT function in complex multicellular environments. Such approaches appear to be essential to determine when TNTs act as protective potential and when they become vectors of pathology. Ultimately, a deeper mechanistic understanding of TNT regulation may enable strategies that selectively amplify their regenerative potential while limiting their contribution to disease propagation, establishing TNTs as both biomarkers and actionable targets in the diagnosis and treatment of complex disorders.

## Figures and Tables

**Table 1:** Minimal operational checklist for validation and recommended experimental criteria for bona fide TNT identification *.

Category	Criterion/Recommendation	Purpose
Structural validation	Live-cell imaging with membranous bridge physically connecting two non-adjacent cells	Excludes fixation artifacts and static membrane remnants
	Demonstration that the structure is suspended above the substratum (e.g., z-stacks, orthogonal views)	Distinguishes TNTs from substrate-attached protrusions
Connection evidence	Evidence of membrane dye and/or cytoplasmic markers	Confirms direct cell–cell connection
Exclusion of extracellular routes	Conditioned medium transfer controls	Rules out diffusion or secreted factors
	Transwell or physical separation assays	Excludes contact-independent transfer
	EV-depleted media or EV-inhibition strategies	Discriminates TNT-mediated transfer from EV uptake
Perturbation tests	Cytoskeletal or signaling perturbations that reduce TNT formation or cargo transfer	Demonstrates functional dependence on TNTs
Imaging requirements (minimum reporting)	Time-lapse duration sufficient to capture TNT formation and cargo movement	Avoids misclassification from static images
	Frame rate appropriate to cargo dynamics	Ensures temporal resolution
	Z-stacks rather than single-plane imaging	Confirms suspended architecture
	Controlled temperature, CO_2_, and humidity	Preserves TNT stability
	High-NA objective (≥1.3 preferred)	Resolves submicron structures
Quantitative metrics (recommended)	TNT density per cell or per field	Enables comparisons across conditions
	TNT length and thickness distributions (thin vs. thick).	Captures structural heterogeneity
	TNT lifetime	Reflects stability and functional relevance
	Frequency of cell–cell contacts via TNTs	Measures network connectivity
	Cargo flux rate (e.g., particles/min or µm/s)	Quantifies transport efficiency
	Fraction of donor cells successfully transferring cargo	Links TNT formation to functional outcome

* Not all TNT studies satisfy the criteria listed above, but the checklist provides a standardized framework for rigor and reproducibility in TNT research. Abbreviations: TNT: tunneling nanotube; EV: extracellular vesicle; NA: numerical aperture of the objective lens. *Thin* vs. *thick TNTs* refer to protrusions with distinct diameters, often associated with differences in cytoskeletal composition and transport capacity. *Cargo flux rate* refers to the rate of particle or signal transfer through TNTs.

**Table 2:** Experimental strategies and recommended readouts for mechanistic, causal, and quantitative analysis of TNTs *.

Aspect	Recommended Approach	Expected Readout/Interpretation
Structural classification (thin vs. thick TNTs)	Dual labeling of F-actin and microtubules (e.g., phalloidin for F-actin + tubulin for microtubules); diameter estimation using high-NA objectives or super-resolution imaging	Actin-only TNTs classified as thin; actin + microtubule-containing TNTs classified as thick
Functional classification (cargo competence)	Live-cell imaging of cargo transfer (ions, vesicles, mitochondria)	Distinguish signaling-only TNTs from cargo-competent TNTs; recommend reporting functional class independently of structure
Actin-dependent formation	Low-dose actin polymerization inhibitors (e.g., cytochalasin D, latrunculin)	Reduced TNT formation or elongation; caution against cytotoxic doses
Contractility and force generation	Myosin II or contractility modulators (e.g., blebbistatin, ROCK inhibitors)	Altered TNT stability or lifetime
Microtubule contribution (thick TNTs)	Microtubule disruptors (e.g., nocodazole, colchicine)	Selective impairment of thick TNT stability and long-range cargo transport
Membrane contribution	Cholesterol depletion or enrichment	Changes in TNT initiation, frequency, and stability
Regulatory pathways	Perturbation of small GTPases or candidate regulators (pharmacological or genetic)	Reduced TNT formation or transfer efficiency
Causal validation	Inhibition/knockdown followed by rescue or re-expression	Rescue of TNT formation or cargo transfer supports pathway specificity
Quantitative metrics	TNT density per cell, length distribution, lifetime, thickness category, contact frequency	Enables comparison across conditions and astrocyte states
Cargo flux	Time-resolved tracking of cargo movement	Compute transfer rate (µm/min), transfer probability, donor efficiency
Mandatory controls	Cell viability, membrane and cytoskeleton integrity	Distinguish TNT-specific effects from general cell viability changes

* Again, not all TNT studies satisfy the aspects listed above, but together they provide recommended approaches for TNT analysis. Abbreviations: TNT: tunneling nanotube; NA: numerical aperture of the objective lens; ROCK: Rho-associated protein kinase. *Thin* vs. *thick TNTs* refer to TNT subtypes with distinct diameters and cytoskeletal composition, often associated with differences in transport capacity. *Cargo competence* refers to the ability of TNTs to transfer material cargo, as opposed to solely mediating signaling events. *Donor efficiency* refers to the fraction of donor cells that successfully transfer cargo to connected recipient cells. *Rescue experiments* refer to re-expression or restoration of a perturbed factor following inhibition or knockdown to demonstrate pathway specificity.

## Data Availability

No new data were created or analyzed in this study.

## Abbreviations

The following abbreviations are used in this manuscript:

2D	Two-dimensional
3D	Three-dimensional
AD	Alzheimer’s disease
AFM	Atomic force microscopy
AKT	Protein kinase B
ATP	Adenosine triphosphate
Aβ	β-amyloid
CNS	Central nervous system
EV	Extracellular vesicle
F-actin	Actin filaments
FAK	Focal adhesion kinase
GBM	Glioblastoma
HD	Huntington’s disease
HGF	Hepatocyte growth factor
I-BAR	Inverse Bin–Amphiphysin–Rvs domain family
IL-1β	Interleukin-1 β
IL-6	Interleukin-6
IL-8	Interleukin-8
mHTT	Mutant huntingtin protein
MSC	Mesenchymal stem cell
mTOR	Mammalian Target of Rapamycin
NMeds	Nanomedicines
OT	Optical tweezers
PD	Parkinson’s disease
PI3K	Phosphoinositide 3-kinase
PLGA	Poly(lactic-co-glycolic acid)
QD	Quantum dots
ROS	Reactive oxygen species
SASP	Senescence-associated secretory phenotype
SIRT1	Sirtuin 1
TiO_2_	Titanium Dioxide
TME	Tumor microenvironment
TNF-α	Tumor necrosis factor-α
TNT	Tunneling nanotube
VEGF	Vascular endothelial growth factor
wtHTT	Wild-type huntingtin protein
YAP	Yes-associated protein
α-syn	α-synuclein

## References

[1] Caire M.J., Reddy V., Varacallo M.A. (2025). Physiology, Synapse.

[2] Olivera A. (2023). Types of Cell Communication: Autocrine, Paracrine, and Endocrine Signaling. Bio. Med..

[3] Durkee C.A., Araque A. (2019). Diversity and Specificity of Astrocyte-neuron Communication. Neuroscience.

[4] Diniz L.P., Almeida J.C., Tortelli V., Vargas Lopes C., Setti-Perdigão P., Stipursky J., Kahn S.A., Romão L.F., de Miranda J., Alves-Leon S.V. (2012). Astrocyte-induced synaptogenesis is mediated by transforming growth factor β signaling through modulation of D-serine levels in cerebral cortex neurons. J. Biol. Chem..

[5] Diniz L.P., Matias I.C., Garcia M.N., Gomes F.C. (2014). Astrocytic control of neural circuit formation: Highlights on TGF-beta signaling. Neurochem. Int..

[6] Dolmans W.M., van Loon A.M., van den Akker R., Mulder D.W., Shao J.F., Mbena E., Mtey P. (1989). Prevalence of HIV-1 antibody among groups of patients and healthy subjects from a rural and urban population in the Mwanza region, Tanzania. Aids.

[7] Rustom A., Saffrich R., Markovic I., Walther P., Gerdes H.-H. (2004). Nanotubular Highways for Intercellular Organelle Transport. Science.

[8] Resnik N., Erman A., Veranic P., Kreft M.E. (2019). Triple labelling of actin filaments, intermediate filaments and microtubules for broad application in cell biology: Uncovering the cytoskeletal composition in tunneling nanotubes. Histochem. Cell Biol..

[9] Pisani F., Castagnola V., Simone L., Loiacono F., Svelto M., Benfenati F. (2022). Role of pericytes in blood-brain barrier preservation during ischemia through tunneling nanotubes. Cell Death Dis..

[10] Raghavan A., Kashyap R., Sreedevi P., Jos S., Chatterjee S., Alex A., D’Souza M.N., Giridharan M., Muddashetty R., Manjithaya R. (2024). Astroglia proliferate upon the biogenesis of tunneling nanotubes via alpha-synuclein dependent transient nuclear translocation of focal adhesion kinase. iScience.

[11] Valdebenito S., Malik S., Luu R., Loudig O., Mitchell M., Okafo G., Bhat K., Prideaux B., Eugenin E.A. (2021). Tunneling nanotubes, TNT, communicate glioblastoma with surrounding non-tumor astrocytes to adapt them to hypoxic and metabolic tumor conditions. Sci. Rep..

[12] Kotarba S., Kozlowska M., Scios M., Saramowicz K., Barczuk J., Granek Z., Siwecka N., Wiese W., Golberg M., Galita G. (2024). Potential Mechanisms of Tunneling Nanotube Formation and Their Role in Pathology Spread in Alzheimer’s Disease and Other Proteinopathies. Int. J. Mol. Sci..

[13] Dilna A., Deepak K.V., Damodaran N., Kielkopf C.S., Kagedal K., Ollinger K., Nath S. (2021). Amyloid-beta induced membrane damage instigates tunneling nanotube-like conduits by p21-activated kinase dependent actin remodulation. Biochim. Biophys. Acta Mol. Basis Dis..

[14] Rostami J., Holmqvist S., Lindstrom V., Sigvardson J., Westermark G.T., Ingelsson M., Bergstrom J., Roybon L., Erlandsson A. (2017). Human Astrocytes Transfer Aggregated Alpha-Synuclein via Tunneling Nanotubes. J. Neurosci..

[15] Tardivel M., Begard S., Bousset L., Dujardin S., Coens A., Melki R., Buee L., Colin M. (2016). Tunneling nanotube (TNT)-mediated neuron-to neuron transfer of pathological Tau protein assemblies. Acta Neuropathol. Commun..

[16] Abounit S., Bousset L., Loria F., Zhu S., de Chaumont F., Pieri L., Olivo-Marin J.C., Melki R., Zurzolo C. (2016). Tunneling nanotubes spread fibrillar alpha-synuclein by intercellular trafficking of lysosomes. EMBO J..

[17] Sun X., Wang Y., Zhang J., Tu J., Wang X.J., Su X.D., Wang L., Zhang Y. (2012). Tunneling-nanotube direction determination in neurons and astrocytes. Cell Death Dis..

[18] Dagar S., Subramaniam S. (2023). Tunneling Nanotube: An Enticing Cell-Cell Communication in the Nervous System. Biology.

[19] Chen Y., Xiao D., Li X. (2024). The role of mitochondrial transfer via tunneling nanotubes in the central nervous system: A review. Medicine.

[20] Khattar K.E., Safi J., Rodriguez A.M., Vignais M.L. (2022). Intercellular Communication in the Brain through Tunneling Nanotubes. Cancers.

[21] Drab M., Kralj-Iglič V., Resnik N., Kreft M.E., Veranič P., Iglič A., Iglič A., Rappolt M., Losada Perez P. (2023). Chapter Four—Formation principles of tunneling nanotubes. Advances in Biomembranes and Lipid Self-Assembly.

[22] Dagar S., Pathak D., Oza H.V., Mylavarapu S.V.S. (2021). Tunneling nanotubes and related structures: Molecular mechanisms of formation and function. Biochem. J..

[23] Veranic P., Lokar M., Schutz G.J., Weghuber J., Wieser S., Hagerstrand H., Kralj-Iglic V., Iglic A. (2008). Different types of cell-to-cell connections mediated by nanotubular structures. Biophys. J..

[24] Malik S., Eugenin E.A. (2016). Mechanisms of HIV Neuropathogenesis: Role of Cellular Communication Systems. Curr. HIV Res..

[25] Resnik N., Baraga D., Glazar P., Jokhadar Zemljic S., Derganc J., Sepcic K., Veranic P., Kreft M.E. (2022). Molecular, morphological and functional properties of tunnelling nanotubes between normal and cancer urothelial cells: New insights from the in vitro model mimicking the situation after surgical removal of the urothelial tumor. Front. Cell Dev. Biol..

[26] Taiarol L., Formicola B., Fagioli S., Sierri G., D’Aloia A., Kravicz M., Renda A., Viale F., Dal Magro R., Ceriani M. (2021). The 3.0 Cell Communication: New Insights in the Usefulness of Tunneling Nanotubes for Glioblastoma Treatment. Cancers.

[27] Errede M., Mangieri D., Longo G., Girolamo F., de Trizio I., Vimercati A., Serio G., Frei K., Perris R., Virgintino D. (2018). Tunneling nanotubes evoke pericyte/endothelial communication during normal and tumoral angiogenesis. Fluids Barriers CNS.

[28] Drab M., Stopar D., Kralj-Iglic V., Iglic A. (2019). Inception Mechanisms of Tunneling Nanotubes. Cells.

[29] Kabaso D., Bobrovska N., Gozdz W., Gov N., Kralj-Iglic V., Veranic P., Iglic A. (2012). On the role of membrane anisotropy and BAR proteins in the stability of tubular membrane structures. J. Biomech..

[30] Ayala Y.A., Pontes B., Hissa B., Monteiro A.C., Farina M., Moura-Neto V., Viana N.B., Nussenzveig H.M. (2017). Effects of cytoskeletal drugs on actin cortex elasticity. Exp. Cell Res..

[31] Pontes B., Viana N.B., Salgado L.T., Farina M., Moura Neto V., Nussenzveig H.M. (2011). Cell cytoskeleton and tether extraction. Biophys. J..

[32] Soares J., Freitas D.G., LourenÇO P.S., Farias J., Pontes B. (2022). Mechanobiology of the cell surface: Probing its remodeling dynamics using membrane tether pulling assays with optical tweezers. Biocell.

[33] Pontes B., Ayala Y., Fonseca A.C., Romao L.F., Amaral R.F., Salgado L.T., Lima F.R., Farina M., Viana N.B., Moura-Neto V. (2013). Membrane elastic properties and cell function. PLoS ONE.

[34] Soares J., Araujo G.R.S., Santana C., Matias D., Moura-Neto V., Farina M., Frases S., Viana N.B., Romao L., Nussenzveig H.M. (2020). Membrane Elastic Properties During Neural Precursor Cell Differentiation. Cells.

[35] Capobianco D.L., Simone L., Svelto M., Pisani F. (2023). Intercellular crosstalk mediated by tunneling nanotubes between central nervous system cells. What we need to advance. Front. Physiol..

[36] Iglic A., Slivnik T., Kralj-Iglic V. (2007). Elastic properties of biological membranes influenced by attached proteins. J. Biomech..

[37] Perutkova S., Kralj-Iglic V., Frank M., Iglic A. (2010). Mechanical stability of membrane nanotubular protrusions influenced by attachment of flexible rod-like proteins. J. Biomech..

[38] Gov N.S., Gopinathan A. (2006). Dynamics of membranes driven by actin polymerization. Biophys. J..

[39] Pontes B., Monzo P., Gauthier N.C. (2017). Membrane tension: A challenging but universal physical parameter in cell biology. Semin. Cell Dev. Biol..

[40] Gousset K., Marzo L., Commere P.H., Zurzolo C. (2013). Myo10 is a key regulator of TNT formation in neuronal cells. J. Cell Sci..

[41] Wang Y., Cui J., Sun X., Zhang Y. (2011). Tunneling-nanotube development in astrocytes depends on p53 activation. Cell Death Differ..

[42] Kretschmer A., Zhang F., Somasekharan S.P., Tse C., Leachman L., Gleave A., Li B., Asmaro I., Huang T., Kotula L. (2019). Stress-induced tunneling nanotubes support treatment adaptation in prostate cancer. Sci. Rep..

[43] Zhang L., Zhang Y. (2015). Tunneling nanotubes between rat primary astrocytes and C6 glioma cells alter proliferation potential of glioma cells. Neurosci. Bull..

[44] Desir S., Dickson E.L., Vogel R.I., Thayanithy V., Wong P., Teoh D., Geller M.A., Steer C.J., Subramanian S., Lou E. (2016). Tunneling nanotube formation is stimulated by hypoxia in ovarian cancer cells. Oncotarget.

[45] Eugenin E.A., Gaskill P.J., Berman J.W. (2009). Tunneling nanotubes (TNT): A potential mechanism for intercellular HIV trafficking. Commun. Integr. Biol..

[46] Pontes B., Viana N.B., Campanati L., Farina M., Neto V.M., Nussenzveig H.M. (2008). Structure and elastic properties of tunneling nanotubes. Eur. Biophys. J..

[47] Patheja P., Dasgupta R., Dube A., Ahlawat S., Verma R.S., Gupta P.K. (2015). The use of optical trap and microbeam to investigate the mechanical and transport characteristics of tunneling nanotubes in tumor spheroids. J. Biophotonics.

[48] Li A., Han X., Deng L., Wang X. (2022). Mechanical properties of tunneling nanotube and its mechanical stability in human embryonic kidney cells. Front. Cell Dev. Biol..

[49] Sun Y., Zhang H., Zavodnik I.B., Zhao H., Feng X. (2024). Mechanical properties of intercellular tunneling nanotubes formed by different mechanisms. Heliyon.

[50] Wang Y., Han X., Deng L., Wang X. (2024). Tunneling nanotube-transmitted mechanical signal and its cellular response. Biochem. Biophys. Res. Commun..

[51] Pascoal P., Kosanic D., Gjoni M., Vogel H. (2010). Membrane nanotubes drawn by optical tweezers transmit electrical signals between mammalian cells over long distances. Lab Chip.

[52] Zhang H., Xu S., Jeffries G.D.M., Orwar O., Jesorka A. (2013). Artificial nanotube connections and transport of molecular cargo between mammalian cells. Nano Commun. Netw..

[53] Kozintsev A., Sugihara K. (2017). Artificial tubular connections between cells based on synthetic lipid nanotubes. RSC Adv..

[54] Bauer B., Davidson M., Orwar O. (2006). Direct reconstitution of plasma membrane lipids and proteins in nanotube-vesicle networks. Langmuir.

[55] Davidson M., Karlsson M., Sinclair J., Sott K., Orwar O. (2003). Nanotube-vesicle networks with functionalized membranes and interiors. J. Am. Chem. Soc..

[56] Karlsson A., Karlsson R., Karlsson M., Cans A.S., Stromberg A., Ryttsen F., Orwar O. (2001). Networks of nanotubes and containers. Nature.

[57] Karlsson M., Sott K., Davidson M., Cans A.S., Linderholm P., Chiu D., Orwar O. (2002). Formation of geometrically complex lipid nanotube-vesicle networks of higher-order topologies. Proc. Natl. Acad. Sci. USA.

[58] Schanke I.J., Xue L., Spustova K., Gozen I. (2022). Transport among protocells via tunneling nanotubes. Nanoscale.

[59] Pinto G., Saenz-de-Santa-Maria I., Chastagner P., Perthame E., Delmas C., Toulas C., Moyal-Jonathan-Cohen E., Brou C., Zurzolo C. (2021). Patient-derived glioblastoma stem cells transfer mitochondria through tunneling nanotubes in tumor organoids. Biochem. J..

[60] Onfelt B., Nedvetzki S., Benninger R.K., Purbhoo M.A., Sowinski S., Hume A.N., Seabra M.C., Neil M.A., French P.M., Davis D.M. (2006). Structurally distinct membrane nanotubes between human macrophages support long-distance vesicular traffic or surfing of bacteria. J. Immunol..

[61] Lu J., Zheng X., Li F., Yu Y., Chen Z., Liu Z., Wang Z., Xu H., Yang W. (2017). Tunneling nanotubes promote intercellular mitochondria transfer followed by increased invasiveness in bladder cancer cells. Oncotarget.

[62] Zampieri L.X., Silva-Almeida C., Rondeau J.D., Sonveaux P. (2021). Mitochondrial Transfer in Cancer: A Comprehensive Review. Int. J. Mol. Sci..

[63] Soundara Rajan T., Gugliandolo A., Bramanti P., Mazzon E. (2020). Tunneling Nanotubes-Mediated Protection of Mesenchymal Stem Cells: An Update from Preclinical Studies. Int. J. Mol. Sci..

[64] Thayanithy V., Dickson E.L., Steer C., Subramanian S., Lou E. (2014). Tumor-stromal cross talk: Direct cell-to-cell transfer of oncogenic microRNAs via tunneling nanotubes. Transl. Res..

[65] Guan F., Wu X., Zhou J., Lin Y., He Y., Fan C., Zeng Z., Xiong W. (2024). Mitochondrial transfer in tunneling nanotubes-a new target for cancer therapy. J. Exp. Clin. Cancer Res..

[66] Pittenger M.F., Discher D.E., Peault B.M., Phinney D.G., Hare J.M., Caplan A.I. (2019). Mesenchymal stem cell perspective: Cell biology to clinical progress. npj Regen. Med..

[67] Figeac F., Lesault P.F., Le Coz O., Damy T., Souktani R., Trebeau C., Schmitt A., Ribot J., Mounier R., Guguin A. (2014). Nanotubular crosstalk with distressed cardiomyocytes stimulates the paracrine repair function of mesenchymal stem cells. Stem Cells.

[68] Babenko V.A., Silachev D.N., Popkov V.A., Zorova L.D., Pevzner I.B., Plotnikov E.Y., Sukhikh G.T., Zorov D.B. (2018). Miro1 Enhances Mitochondria Transfer from Multipotent Mesenchymal Stem Cells (MMSC) to Neural Cells and Improves the Efficacy of Cell Recovery. Molecules.

[69] Tenchov R., Sasso J.M., Wang X., Zhou Q.A. (2024). Aging Hallmarks and Progression and Age-Related Diseases: A Landscape View of Research Advancement. ACS Chem. Neurosci..

[70] Pessoa B., Hayashide L.d.S., Dias G., Pontes B., Pinto R.S., Diniz L.P. (2026). Senescent Astrocytes: A New Player in Brain Aging and Cognitive Decline. Brain Sci..

[71] Won W., Bhalla M., Lee J.H., Lee C.J. (2025). Astrocytes as Key Regulators of Neural Signaling in Health and Disease. Annu. Rev. Neurosci..

[72] Villablanca C., Vidal R., Gonzalez-Billault C. (2023). Are cytoskeleton changes observed in astrocytes functionally linked to aging?. Brain Res. Bull..

[73] Diniz L.P., Araujo A.P.B., Carvalho C.F., Matias I., de Sa Hayashide L., Marques M., Pessoa B., Andrade C.B.V., Vargas G., Queiroz D.D. (2024). Accumulation of damaged mitochondria in aging astrocytes due to mitophagy dysfunction: Implications for susceptibility to mitochondrial stress. Biochim. Biophys. Acta Mol. Basis Dis..

[74] Marques M., Hayashide L.S., Amorim P., Fernandes B.M., Araujo A.P.B., Messor D.F., Leocadio V.E., Pessoa B., Correa J., Villablanca C. (2025). Doxorubicin Induces a Senescent Phenotype in Murine and Human Astrocytes. J. Neurochem..

[75] Mijit M., Caracciolo V., Melillo A., Amicarelli F., Giordano A. (2020). Role of p53 in the Regulation of Cellular Senescence. Biomolecules.

[76] Verma D.K., Seo B.A., Ghosh A., Ma S.X., Hernandez-Quijada K., Andersen J.K., Ko H.S., Kim Y.H. (2021). Alpha-Synuclein Preformed Fibrils Induce Cellular Senescence in Parkinson’s Disease Models. Cells.

[77] Lurette O., Martin-Jimenez R., Khan M., Sheta R., Jean S., Schofield M., Teixeira M., Rodriguez-Aller R., Perron I., Oueslati A. (2023). Aggregation of alpha-synuclein disrupts mitochondrial metabolism and induce mitophagy via cardiolipin externalization. Cell Death Dis..

[78] Araujo A.P.B., Vargas G., Hayashide L.S., Matias I., Andrade C.B.V., de Carvalho J.J., Gomes F.C.A., Diniz L.P. (2024). Aging promotes an increase in mitochondrial fragmentation in astrocytes. Front. Cell. Neurosci..

[79] Mulica P., Grunewald A., Pereira S.L. (2021). Astrocyte-Neuron Metabolic Crosstalk in Neurodegeneration: A Mitochondrial Perspective. Front. Endocrinol..

[80] Matias I., Diniz L.P., Damico I.V., Araujo A.P.B., Neves L.D.S., Vargas G., Leite R.E.P., Suemoto C.K., Nitrini R., Jacob-Filho W. (2022). Loss of lamin-B1 and defective nuclear morphology are hallmarks of astrocyte senescence in vitro and in the aging human hippocampus. Aging Cell.

[81] Ranzinger J., Rustom A., Abel M., Leyh J., Kihm L., Witkowski M., Scheurich P., Zeier M., Schwenger V. (2011). Nanotube action between human mesothelial cells reveals novel aspects of inflammatory responses. PLoS ONE.

[82] Ziegler D.V., Wiley C.D., Velarde M.C. (2015). Mitochondrial effectors of cellular senescence: Beyond the free radical theory of aging. Aging Cell.

[83] Raghavan A., Rao P., Neuzil J., Pountney D.L., Nath S. (2021). Oxidative stress and Rho GTPases in the biogenesis of tunnelling nanotubes: Implications in disease and therapy. Cell. Mol. Life Sci..

[84] Lin X., Wang W., Chang X., Chen C., Guo Z., Yu G., Shao W., Wu S., Zhang Q., Zheng F. (2024). ROS/mtROS promotes TNTs formation via the PI3K/AKT/mTOR pathway to protect against mitochondrial damages in glial cells induced by engineered nanomaterials. Part. Fibre Toxicol..

[85] Whitehead J., Zhang J., Harvestine J.N., Kothambawala A., Liu G.Y., Leach J.K. (2020). Tunneling nanotubes mediate the expression of senescence markers in mesenchymal stem/stromal cell spheroids. Stem Cells.

[86] Chen C., Zhou M., Ge Y., Wang X. (2020). SIRT1 and aging related signaling pathways. Mech. Ageing Dev..

[87] Wang Q., Zhu K., Zhang A. (2024). SIRT1-mediated tunnelling nanotubes may be a potential intervention target for arsenic-induced hepatocyte senescence and liver damage. Sci. Total Environ..

[88] Sun Y., Yu Y., Ma S., Liao C., Yang J., Lyu Y., Zhang X., Zhang J., Tian W., Liao L. (2024). Nanotube topography rejuvenates the senescence of mesenchymal stem cells by activating YAP signalling. J. Mater. Chem. B.

[89] Bagheri-Mohammadi S. (2021). Stem cell-based therapy as a promising approach in Alzheimer’s disease: Current perspectives on novel treatment. Cell Tissue Bank..

[90] Wang X., Wang W., Li L., Perry G., Lee H.G., Zhu X. (2014). Oxidative stress and mitochondrial dysfunction in Alzheimer’s disease. Biochim. Biophys. Acta.

[91] Giulian D. (1999). Microglia and the immune pathology of Alzheimer disease. Am. J. Hum. Genet..

[92] Wang X.T., Sun H., Chen N.H., Yuan Y.H. (2021). Tunneling nanotubes: A novel pharmacological target for neurodegenerative diseases?. Pharmacol. Res..

[93] Malekpour K., Hazrati A., Soudi S., Hashemi S.M. (2023). Mechanisms behind therapeutic potentials of mesenchymal stem cell mitochondria transfer/delivery. J. Control. Release.

[94] Capobianco D.L., De Zio R., Profico D.C., Gelati M., Simone L., D’Erchia A.M., Di Palma F., Mormone E., Bernardi P., Sbarbati A. (2024). Human neural stem cells derived from fetal human brain communicate with each other and rescue ischemic neuronal cells through tunneling nanotubes. Cell Death Dis..

[95] Chastagner P., Loria F., Vargas J.Y., Tois J., Diamond M.I., Okafo G., Brou C., Zurzolo C. (2020). Fate and propagation of endogenously formed Tau aggregates in neuronal cells. EMBO Mol. Med..

[96] Engelke A.D., Gonsberg A., Thapa S., Jung S., Ulbrich S., Seidel R., Basu S., Multhaup G., Baier M., Engelhard M. (2018). Dimerization of the cellular prion protein inhibits propagation of scrapie prions. J. Biol. Chem..

[97] Vanni I., Pirisinu L., Acevedo-Morantes C., Kamali-Jamil R., Rathod V., Di Bari M.A., D’Agostino C., Marcon S., Esposito E., Riccardi G. (2020). Isolation of infectious, non-fibrillar and oligomeric prions from a genetic prion disease. Brain.

[98] Gousset K., Schiff E., Langevin C., Marijanovic Z., Caputo A., Browman D.T., Chenouard N., de Chaumont F., Martino A., Enninga J. (2009). Prions hijack tunnelling nanotubes for intercellular spread. Nat. Cell Biol..

[99] Rouvinski A., Karniely S., Kounin M., Moussa S., Goldberg M.D., Warburg G., Lyakhovetsky R., Papy-Garcia D., Kutzsche J., Korth C. (2014). Live imaging of prions reveals nascent PrPSc in cell-surface, raft-associated amyloid strings and webs. J. Cell Biol..

[100] Gousset K., Zurzolo C. (2009). Tunnelling nanotubes: A highway for prion spreading?. Prion.

[101] Marzo L., Gousset K., Zurzolo C. (2012). Multifaceted roles of tunneling nanotubes in intercellular communication. Front. Physiol..

[102] Costanzo M., Abounit S., Marzo L., Danckaert A., Chamoun Z., Roux P., Zurzolo C. (2013). Transfer of polyglutamine aggregates in neuronal cells occurs in tunneling nanotubes. J. Cell Sci..

[103] Ren P.H., Lauckner J.E., Kachirskaia I., Heuser J.E., Melki R., Kopito R.R. (2009). Cytoplasmic penetration and persistent infection of mammalian cells by polyglutamine aggregates. Nat. Cell Biol..

[104] Saudou F., Finkbeiner S., Devys D., Greenberg M.E. (1998). Huntingtin acts in the nucleus to induce apoptosis but death does not correlate with the formation of intranuclear inclusions. Cell.

[105] Sharma M., Subramaniam S. (2019). Rhes travels from cell to cell and transports Huntington disease protein via TNT-like protrusion. J. Cell Biol..

[106] Spillantini M.G., Crowther R.A., Jakes R., Hasegawa M., Goedert M. (1998). alpha-Synuclein in filamentous inclusions of Lewy bodies from Parkinson’s disease and dementia with lewy bodies. Proc. Natl. Acad. Sci. USA.

[107] Sarkari A., Lou E. (2024). Do tunneling nanotubes drive chemoresistance in solid tumors and other malignancies?. Biochem. Soc. Trans..

[108] Ottonelli I., Caraffi R., Tosi G., Vandelli M.A., Duskey J.T., Ruozi B. (2022). Tunneling Nanotubes: A New Target for Nanomedicine?. Int. J. Mol. Sci..

[109] Duskey J.T., Belletti D., Pederzoli F., Vandelli M.A., Forni F., Ruozi B., Tosi G. (2017). Current Strategies for the Delivery of Therapeutic Proteins and Enzymes to Treat Brain Disorders. Int. Rev. Neurobiol..

[110] Righeschi C., Coronnello M., Mastrantoni A., Isacchi B., Bergonzi M.C., Mini E., Bilia A.R. (2014). Strategy to provide a useful solution to effective delivery of dihydroartemisinin: Development, characterization and in vitro studies of liposomal formulations. Colloids Surf. B Biointerfaces.

[111] Duskey J.T., Ottonelli I., Rinaldi A., Parmeggiani I., Zambelli B., Wang L.Z., Prud’homme R.K., Vandelli M.A., Tosi G., Ruozi B. (2021). Tween^®^ Preserves Enzyme Activity and Stability in PLGA Nanoparticles. Nanomaterials.

[112] Cao S.J., Xu S., Wang H.M., Ling Y., Dong J., Xia R.D., Sun X.H. (2019). Nanoparticles: Oral Delivery for Protein and Peptide Drugs. AAPS PharmSciTech.

[113] Pederzoli F., Ruozi B., Duskey J., Hagmeyer S., Sauer A.K., Grabrucker S., Coelho R., Oddone N., Ottonelli I., Daini E. (2019). Nanomedicine Against Abeta Aggregation by beta-Sheet Breaker Peptide Delivery: In Vitro Evidence. Pharmaceutics.

[114] Han X., Su R., Huang X., Wang Y., Kuang X., Zhou S., Liu H. (2019). Triphenylphosphonium-modified mitochondria-targeted paclitaxel nanocrystals for overcoming multidrug resistance. Asian J. Pharm. Sci..

[115] Herrada-Manchon H., Celada L., Rodriguez-Gonzalez D., Alejandro Fernandez M., Aguilar E., Chiara M.D. (2021). Three-dimensional bioprinted cancer models: A powerful platform for investigating tunneling nanotube-like cell structures in complex microenvironments. Mater. Sci. Eng. C Mater. Biol. Appl..

[116] He K., Luo W., Zhang Y., Liu F., Liu D., Xu L., Qin L., Xiong C., Lu Z., Fang X. (2010). Intercellular transportation of quantum dots mediated by membrane nanotubes. ACS Nano.

[117] Mittal R., Karhu E., Wang J.S., Delgado S., Zukerman R., Mittal J., Jhaveri V.M. (2019). Cell communication by tunneling nanotubes: Implications in disease and therapeutic applications. J. Cell. Physiol..

[118] Epperla C.P., Mohan N., Chang C.W., Chen C.C., Chang H.C. (2015). Nanodiamond-Mediated Intercellular Transport of Proteins through Membrane Tunneling Nanotubes. Small.

[119] Rehberg M., Nekolla K., Sellner S., Praetner M., Mildner K., Zeuschner D., Krombach F. (2016). Intercellular Transport of Nanomaterials is Mediated by Membrane Nanotubes In Vivo. Small.

[120] Franco S., Noureddine A., Guo J., Keth J., Paffett M.L., Brinker C.J., Serda R.E. (2020). Direct Transfer of Mesoporous Silica Nanoparticles between Macrophages and Cancer Cells. Cancers.

[121] Tosi G., Costantino L., Rivasi F., Ruozi B., Leo E., Vergoni A.V., Tacchi R., Bertolini A., Vandelli M.A., Forni F. (2007). Targeting the central nervous system: In vivo experiments with peptide-derivatized nanoparticles loaded with Loperamide and Rhodamine-123. J. Control. Release.

[122] Salvalaio M., Rigon L., Belletti D., D’Avanzo F., Pederzoli F., Ruozi B., Marin O., Vandelli M.A., Forni F., Scarpa M. (2016). Targeted Polymeric Nanoparticles for Brain Delivery of High Molecular Weight Molecules in Lysosomal Storage Disorders. PLoS ONE.

[123] Kristl J., Plajnsek K.T., Kreft M.E., Jankovic B., Kocbek P. (2013). Intracellular trafficking of solid lipid nanoparticles and their distribution between cells through tunneling nanotubes. Eur. J. Pharm. Sci..

[124] Qin H., Jiang Y., Zhang J., Deng C., Zhong Z. (2019). Oncoprotein Inhibitor Rigosertib Loaded in ApoE-Targeted Smart Polymersomes Reveals High Safety and Potency against Human Glioblastoma in Mice. Mol. Pharm..

[125] Ouyang J., Jiang Y., Deng C., Zhong Z., Lan Q. (2021). Doxorubicin Delivered via ApoE-Directed Reduction-Sensitive Polymersomes Potently Inhibit Orthotopic Human Glioblastoma Xenografts in Nude Mice. Int. J. Nanomed..

[126] Costa P.M., Cardoso A.L., Mendonca L.S., Serani A., Custodia C., Conceicao M., Simoes S., Moreira J.N., Pereira de Almeida L., Pedroso de Lima M.C. (2013). Tumor-targeted Chlorotoxin-coupled Nanoparticles for Nucleic Acid Delivery to Glioblastoma Cells: A Promising System for Glioblastoma Treatment. Mol. Ther. Nucleic Acids.

[127] Cohen-Inbar O., Zaaroor M. (2016). Glioblastoma multiforme targeted therapy: The Chlorotoxin story. J. Clin. Neurosci..

[128] Notario Manzano R., Chaze T., Rubinstein E., Penard E., Matondo M., Zurzolo C., Brou C. (2024). Proteomic landscape of tunneling nanotubes reveals CD9 and CD81 tetraspanins as key regulators. eLife.

